# Regulation of mammalian transcription and splicing by Nuclear RNAi

**DOI:** 10.1093/nar/gkv1305

**Published:** 2015-11-26

**Authors:** Roya Kalantari, Cheng-Ming Chiang, David R. Corey

**Affiliations:** 1 Departments of Pharmacology & Biochemistry, University of Texas Southwestern Medical Center, 6001 Forest Park Road, Dallas, TX 75390-9041, USA; 2 Simmons Comprehensive Cancer Center, University of Texas Southwestern Medical Center, 6001 Forest Park Road, Dallas, TX 75390-8807, USA

## Abstract

RNA interference (RNAi) is well known as a mechanism for controlling mammalian mRNA translation in the cytoplasm, but what would be the consequences if it also functions in cell nuclei? Although RNAi has also been found in nuclei of plants, yeast, and other organisms, there has been relatively little progress towards understanding the potential involvement of mammalian RNAi factors in nuclear processes including transcription and splicing. This review summarizes evidence for mammalian RNAi factors in cell nuclei and mechanisms that might contribute to the control of gene expression. When RNAi factors bind small RNAs, they form ribonucleoprotein complexes that can be selective for target sequences within different classes of nuclear RNA substrates. The versatility of nuclear RNAi may supply a previously underappreciated layer of regulation to transcription, splicing, and other nuclear processes.

## INTRODUCTION

The nucleus is a hub of RNA synthesis, processing and regulation (1–3). Transcription and splicing occur in cell nuclei and understanding mammalian gene expression requires detailed knowledge regarding how these processes are regulated. Because both transcription and splicing involve RNA, any mechanism that drives the recognition of RNA target sequences might affect their outcome.

RNA interference (RNAi) provides a mechanism for selective recognition of RNA sequences. Unlike protein transcription factors that are characterized by relatively limited selectivity for target sites and relative difficulty evolving new selectivities, recognition of RNA by small RNAs would exploit the high specificity of Watson–Crick base-pairing and the potential for developing new selectivities by nucleotide mutation. The potential evolutionary advantages of RNA recognition in cell nuclei appear compelling, but how might it be achieved?

RNAi is well known as a regulatory mechanism for controlling gene expression in cell cytoplasm. If the protein machinery and small RNAs responsible for cytoplasmic RNAi were also found in mammalian cell nuclei, it is possible to imagine these factors promoting recognition of nascent transcripts, splice junctions, and other regulatory control points. Such recognition would constitute a previously unappreciated level of regulatory control over gene expression.

Our goal is to describe the current state of research into mammalian nuclear RNAi. We begin by briefly addressing nuclear RNAi in non-mammalian organisms and by addressing the origins of controversy and skepticism towards the existence of RNAi in mammalian somatic cells. We describe reports of nuclear RNA affecting gene repression, gene activation, miRNA function, and gene splicing.

## CYTOPLASMIC RNAi

**Figure 1.**
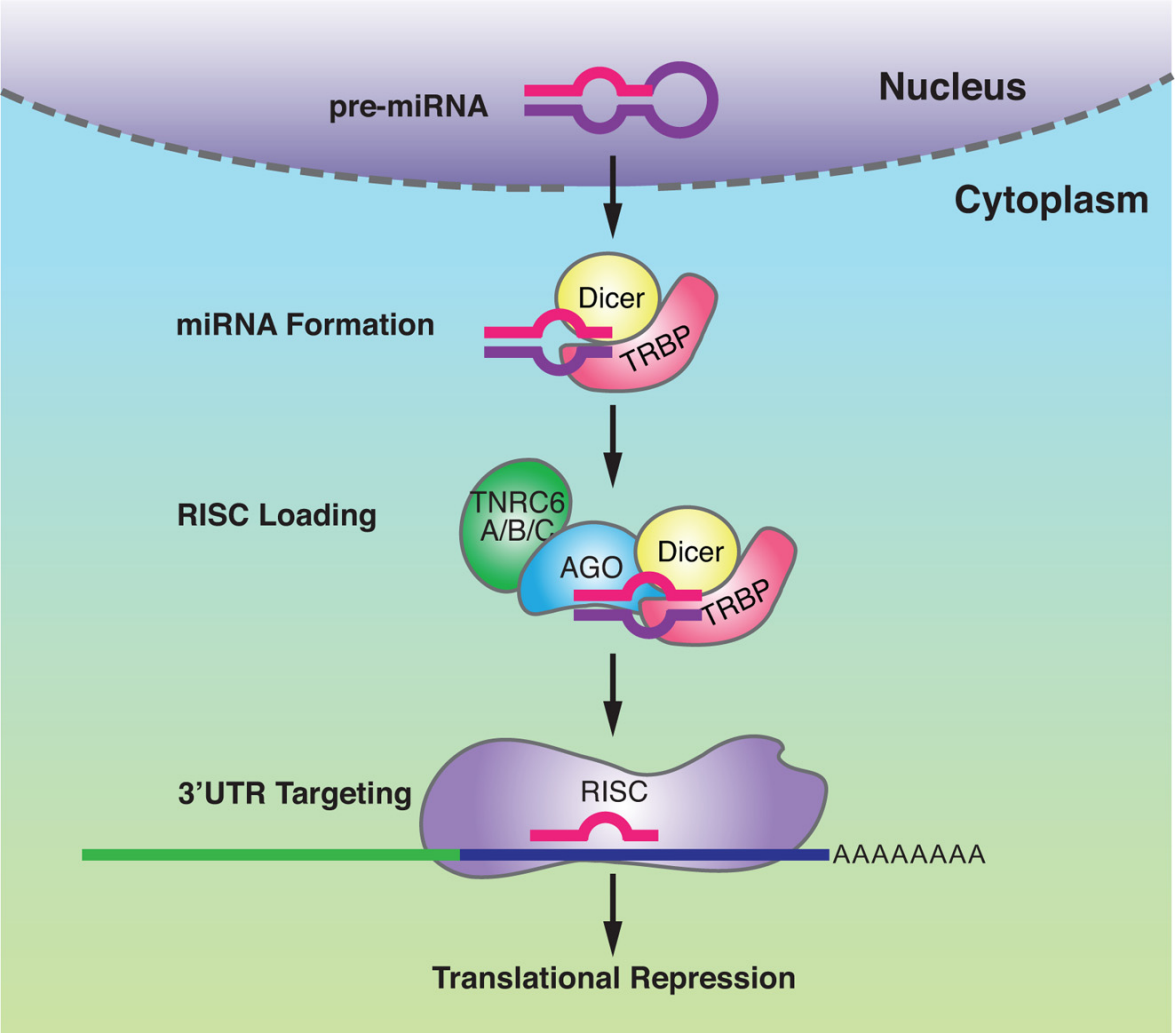
RNA Interference in the cytoplasm of mammalian cells. Pre-miRNAs are transcribed in the nucleus. Dicer and TRBP bind to pre-miRNAs and Dicer cleaves them to form mature, double-stranded miRNAs. The miRNAs are then loaded onto AGO proteins, which along with TNRC6 proteins form the RISC complex. The passenger strand of the miRNA is removed to form an active complex which can then target and bind to the 3′ untranslated regions of mRNAs. This binding leads to translational repression.

RNAi is a powerful mechanism for using small RNAs to control protein translation in the cytoplasm of mammalian cells (4,5). Naturally occurring microRNAs recognize sequences within 3′-untranslated regions to control translation (6) (Figure 1). Synthetic duplex RNAs that are fully complementary to targets within mRNA are widely used to reduce gene expression in the laboratory (7).

The small RNAs are typically duplex RNAs that include a guide strand complementary to the cellular RNA target sequence. During RNAi the guide strand RNA is loaded into an RNA-induced silencing complex (RISC) consisting of protein factors including Argonaute 2 (AGO2) (8–10) and TRNC6A (11,12). The guide strand can be supplied by a synthetic RNA duplex (7), as a chemically stabilized single-strand (13), or can be naturally expressed within a cell as microRNA (miRNA) (14–16).

There are four AGO proteins in mammalian cells (AGO1–4) (17). AGO2 is the central RNAi factor involved in post-transcriptional RNA silencing and is the only AGO variant capable of catalytic cleavage of RNA substrates (8–10). Roles for AGO1, AGO3 and AGO4 are not as well defined but they can bind to RNA, and AGO1 and AGO3 can participate in gene silencing by RNAs targeted to 3′-untranslated regions in the absence of AGO2 (18). TNRC6 family members are the mammalian homologs of the *Drosophila* protein GW182 (19,20). TNRC6 family members associate with AGO (21) and with cytoplasmic P-bodies (19).

The guide strand RNA directs the complex to target sequences through Watson–Crick base-pairing (22). Recognition by Watson–Crick pairing is predictable, and diverse guide strands can participate in ribonucleoprotein complex formation. These features make RISC programmable—depending on the sequence of the guide strand RNA, different cellular RNA sequences can be targeted.

The protein domains of RISC serve multiple functions. Single-stranded RNA is subject to digestion by nucleases and its interaction with proteins helps ensure its survival. The guide RNA binds directly to AGO2 (23–25). The orientation of binding displays the RNA seed sequence in an ideal position to nucleate binding to target RNA (26). When the central portion of the guide RNA is fully complementary to its target, AGO2 can cleave the target RNA (10). Binding by AGO2 masks the negative charge of the RNA backbone, facilitating recognition of complementary RNA and accelerating searching and identification of complementary sequences (24,25).

## NUCLEAR RNAi IN NON-MAMMALIAN SYSTEMS

The potential for RNA to induce sequence-specific changes in the nucleus of non-mammalian organisms has been recognized for over two decades and has been extensively reviewed (27–31).

**Figure 2.**
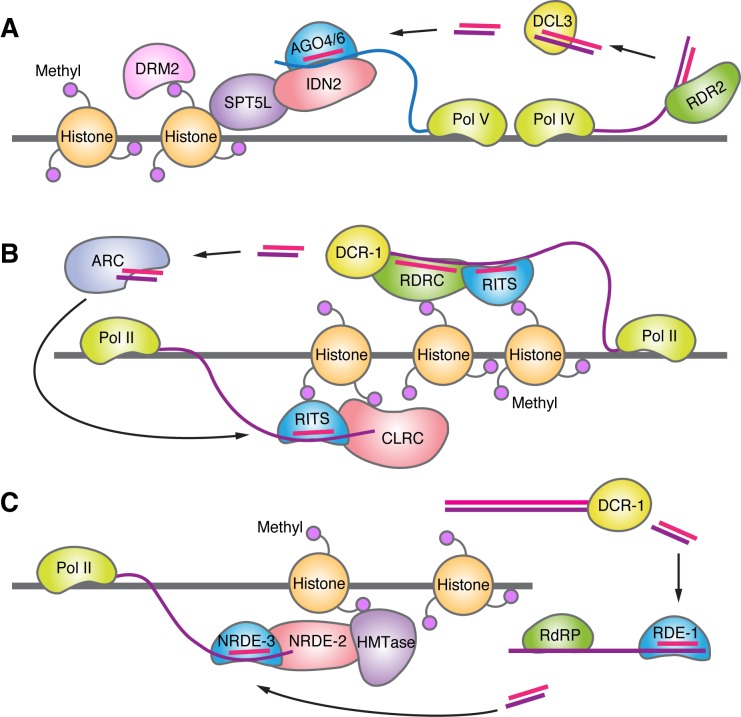
RNA interference in non-mammalian nuclei. (**A**) DNA methylation in plants. Pol IV transcribes long RNA that RDR2 uses as a template to form double-stranded RNA. DCL3 processes this transcript to create double-stranded small RNAs that are loaded onto AGO4 or 6. The small RNA targets AGO4/6 to nascent RNA transcribed by Pol V. The methylation factors IDN2, SPT5L, and DRM2 then localize to the site, causing methylation and silencing. (**B**) Histone methylation in yeast. Pol II transcribes long RNAs that are bound by the RITS complex and RDRC that uses the RNA as a template to form double-stranded RNA. DCR-1 processes this to create double-stranded small RNAs. The small RNAs are loaded onto AGO1 in the RITS complex by the ARC complex. This targets to nascent transcripts along the chromatin. The histone methylation complex CLRC then localizes to the site, causing methylation and silencing. (**C**) Histone methylation in worms. Double-stranded RNA is processed by DCR-1 to form double-stranded small RNAs. These are loaded onto RDE-1, which causes amplification of the small RNA through RdRPs. The small RNAs are then loaded onto NRDE-3, which targets nascent transcripts along the chromatin. NRDE-2 and HMTases then localize to the site, causing methylation and silencing.

Nuclear RNAi was first discovered in plants and shown to induce DNA methylation of sequences at gene promoters (32) (Figure 2A). In this study, viroid RNA replication was necessary for methylation of integrated viral DNA sequences. Expressed small hairpin RNAs that were targeted against plant promoters also induced DNA methylation (32,33). Small RNAs are loaded onto AGO4 (34) and AGO6 (35) and both AGO variants appear to be important for silencing (36,37). The mechanism of recognition for small RNAs involves recognition of noncoding transcripts at gene promoters (38–40). Binding of the small RNA-AGO4 or -AGO6 complex to the nascent RNA then triggers cytosine methylation, possibly through the action of SWI/SNF-mediated chromatin remodeling and DNA methylase-triggered base modification (41).

In the yeast *S. pombe*, RNAi factors modulate gene expression by inducing histone modifications (28,42,43) (Figure 2B). These factors, collectively known as the RNA-Induced Transcriptional Silencing (RITS) complex, consist of AGO1, Chp1, and Tas3 (44). Pol II transcribes RNAs along centromeric repeats that are processed into duplex RNAs by Dicer, or form pri-RNAs that work through a Dicer-independent mechanism (45–47). The small RNAs are loaded onto AGO1 by the AGO siRNA chaperone (ARC) complex in the cytoplasm. Loaded AGO1 then binds to the other members of the RITS complex (27). AGO1 binds to nascent transcripts along centromeric repeats where it tethers the RITS complex along with the Clr4–Rik1–Cul4 (CLRC) complex (48–50). The CLRC is responsible for H3K9 methylation and subsequent transcriptional silencing. H3K9 methylation is recognized by the RNA-directed RNA polymerase complex (RDRC), which nucleates additional siRNA formation and promotes further methylation. A recent study suggests that the Paf1 complex can prevent the recognition of the nascent transcript by duplex RNAs and that synthetic RNAs are much more effective silencing agents in Paf1C mutant *S. pombe* (51).

*C. elegans* also employs RNAi factors to direct H3K9 methylation (27,52) (Figure 2C). In *C. elegans*, double-stranded RNAs are processed by Dicer-1 into siRNAs (53–55). These RNAs are loaded onto an AGO protein, RDE-1, that functions with RNA-dependent RNA polymerases (RdRPs) to amplify the number of small RNAs (56). These RNAs are known as 22G-RNAs. 22G-RNAs are then loaded onto the AGO protein NRDE-3. Loading takes place in the cytoplasm and the NRDE-3/small RNA complex subsequently enters the nucleus (57) and binds to nascent transcripts (58). NRDE-2 binds to NRDE-3 at these sites, promotes H3K9 methylation and silences transcription (58–61).

## MAMMALIAN NUCLEAR RNAi

In spite of the importance of cytoplasmic RNAi in mammalian cells and the role of nuclear RNAi in model organisms, the study of mammalian nuclear RNAi has received little attention. There are several reasons why the study of mammalian nuclear RNAi has lagged behind.

One reason has been confusion over whether RNAi factors were present or active in mammalian somatic cell nuclei. Early work reported that duplex RNAs could not silence expression of introns (62,63). In addition, anecdotal evidence from many laboratories suggested the general silencing of nuclear RNA targets is often difficult to achieve. Microscopy showed localization of AGO2 to cytoplasmic P-bodies and the endoplasmic reticulum, supporting a cytoplasmic role (64–66) and diverting attention further away from any potential nuclear function. A more practical reason is that it can be difficult to purify protein or RNA from cell nuclei in a manner that convincingly eliminates significant amounts of cytoplasmic contaminants.

Interest in mammalian nuclear RNAi was stimulated in 2004 when two reports appeared suggesting that duplex RNAs complementary to gene promoters could repress gene expression in mammalian cells (67,68). These reports implicated DNA methylation and histone modification as causes for silencing. Both reports, however, were subject to criticism in a subsequent paper (69) and one was retracted (70).

Taken together, the mix of negative results, questions about published data, and the focus on cytoplasmic RNAi led to skepticism about whether RNAi factors were present or active within mammalian somatic cell nuclei and whether RNAi could influence nuclear gene expression. It is likely that this skepticism, in combination with the added complexity of working with factors in cell nuclei, slowed entry of researchers into the field and reduced progress.

## EVIDENCE OF RNAi FACTORS IN CELL NUCLEI

Several reports have appeared suggesting that RNAi factors are present in mammalian cell nuclei and can actively process RNA. Two laboratories reported that small RNAs could silence nuclear localized 7SK RNA (71,72). Both Myc-tagged AGO2 (71) and EGFP-tagged AGO2 (72) could be identified in purified cell nuclei. An antibody that recognizes endogenous AGO2 also identified AGO2 in cell nuclei (73).

Fluorescence correlation and fluorescence cross correlation microscopy suggested that AGO2 is loaded in the cytoplasm and subsequently imported into the nucleus (72). Knockdown of Importin 8 reduced the pool of nuclear AGO2 (74,75), suggesting a role in import. The AGO binding partner TNRC6A actively shuttles between the cytoplasm and the nucleus, implying that AGO2 is also imported into nuclei (76). This possibility was supported by the finding that TNRC6A can interact with AGO2 to facilitate the delivery of miRNAs to cell nuclei (77). Most recently, Meister and colleagues have reported that TNRC6 nuclear import is enabled by Importin-β (78). Consistent with a close interaction between AGO2 and TNRC6 (79), nuclear localization of AGO2 and TNRC6 is mutually dependent (77). Dicer has also been observed in cell nuclei, shown to interact with RNA polymerase, and may regulate levels of double-stranded RNA (80). Interestingly, a recent study using novel live cell imaging technology found fast distribution of synthetic siRNA into nuclei after transfection with cationic lipid in a subset of cells (81).

Biochemical studies of cell nuclei run the risk of contamination from cytoplasm, raising the bar for persuasive investigation. For example, AGO2 is partially localized to the endoplasmic reticulum (ER) (64). Since the ER is contiguous with the nuclear membrane and can co-purify with nuclei, it was possible that detection of AGO2 might have been due to ER contamination. Another concern is that RNAs that are assumed to be primarily nuclear can be detected in the cytoplasm (82). Standard cytoplasmic RNAi might cause cleavage of these “nuclear” transcripts and the subsequent transit of fragments into the nucleus might result in confounding conclusions.

Multiple independent methods have recently been used to examine localization of endogenous AGO2 and other RNAi factors by microscopy, cell fractionation, size exclusion chromatography, and activity assays (82). Wide-field immunofluorescence microscopy revealed AGO2 in both cell cytoplasm and nuclei. Using a stringent protocol for purifying nuclei that removed detectable ER contamination (83), western blot analysis of nuclear proteins revealed AGO2, Dicer, TRBP and TNRC6A in cell nuclei. A subsequent study showed that reduced expression of AGO2 affected the nuclear localization of other RNAi factors and that residual AGO2 was disproportionately retained in cell nuclei (84). Recently, a peptide derived from GW182 has been shown to be a powerful tag for isolating RNAi factors and it may be a useful tool for further probing cellular interactions (85).

**Figure 3.**
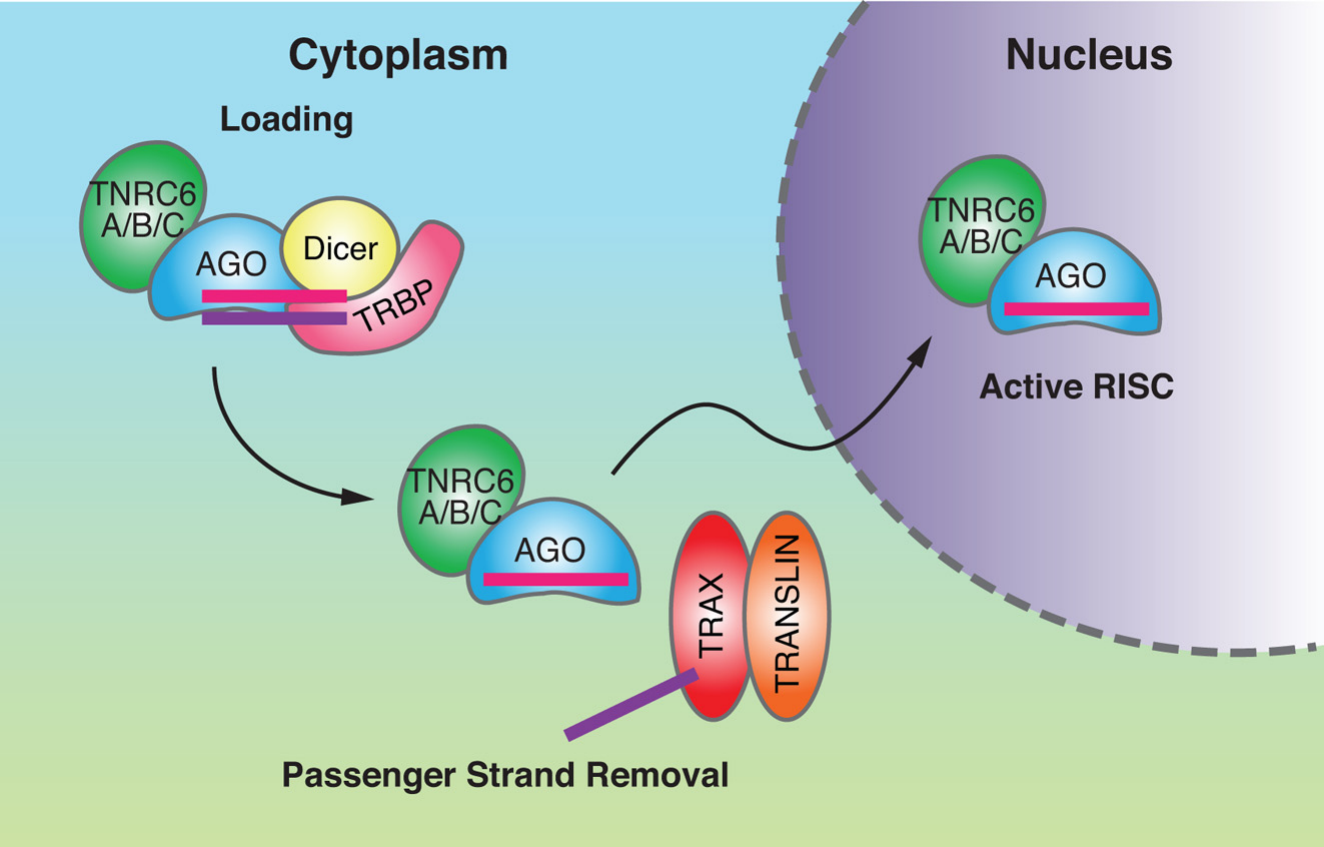
Small RNAs must be loaded in the cytoplasm prior to recognition of targets in cell nuclei (66). Model for transport of loaded RNAs into cell nuclei. Dicer and TRBP load double-stranded small RNAs into AGO2. Trax and Translin aid in passenger strand removal, which activates the complex. AGO2 and TNRC6 then move into the nucleus to target nuclear RNAs.

AGO2-mediated slicer activity in cell nuclei has been observed when RNA duplexes are transfected into cells prior to the activity assays (79). Also consistent with nuclear slicer activity, duplex RNAs can knock down the nuclear localized RNA MALAT1 and eliminate MALAT1 foci. 5′ Rapid amplification of cDNA ends (5′-RACE) fragments that were diagnostic of MALAT-1 cleavage at a predicted RNAi target site were found associated with the chromatin fraction. When an RNA substrate and a complementary duplex RNA were added directly to nuclear extract, however, cleavage of the RNA substrate was not observed. This result was explained by the absence of RNA loading factors in cell nuclei leading to the conclusion that loading takes place in the cytoplasm prior to import into cell nuclei (Figure 3). This result had been previously suggested (72) and is consistent with observations of loading in *C. elegans* (57).

Concurrent with this review, Behlke and Lennox compared silencing of long noncoding RNAs in the nucleus and cytoplasm by duplex RNAs and RNase H-active antisense oligonucleotides (86). This study surveyed effects of dozens of nucleic acid silencing agents on multiple long noncoding RNA targets. They concluded that duplex RNAs could silence RNA targets in cell nuclei. However, compared to silencing triggered by antisense oligonucleotides in the nucleus or RNA-mediated silencing in the cytoplasm, RNA-mediated silencing in cell nuclei was less efficient. More work will be required to determine the mechanism of less efficient RNA-mediated silencing of nuclear targets.

RNAi factors in eukaryotic cell nuclei have also been implicated in DNA damage repair (87–90). At the site of DNA double-strand breaks, small RNAs known as double-strand break induced RNAs (diRNAs) are generated. These RNAs have been shown to be DICER dependent (89,90). The diRNAs target AGO2 to the site of damage along with RAD51, which typically triggers homologous recombination-mediated DNA damage repair (87).

## RNA-MEDIATED CONTROL OF TRANSCRIPTION

### RNA-mediated transcriptional silencing

As noted above, in 2004 two reports appeared suggesting that duplex RNAs that were complementary to mammalian gene promoters could cause DNA methylation and silence gene expression (67,68). While one paper was subsequently retracted (70) the other was not. These results focused attention on the potential for duplex RNA to act in cell nuclei. The duplexes used in these studies were standard siRNAs, implying that RNAi factors might be involved. In 2005, Rossi and colleagues reported that small RNAs targeting the *RASSF1A* gene promoter could induce low levels of DNA methylation and gene silencing (91).

Our laboratory had been studying the ability of peptide nucleic acid oligomers (PNAs) complementary to the progesterone receptor (*PR*) promoter to inhibit expression of chromosomally encoded *PR* expression (92). Because of our experience using the *PR* gene as a model system we evaluated the effect of transfecting duplex RNAs complementary to sequences at the most upstream transcription start site.

Our initial experiments with duplex RNAs complementary to sequences at the *PR* promoter revealed silencing of *PR* RNA and protein expression (93). We did not observe changes in DNA methylation at the *PR* promoter. Kang and co-workers confirmed RNA-mediated gene silencing of *PR* and also androgen receptor (94). The recent publication of a more straightforward assay for quantifying nascent transcript synthesis may supply a better tool than nuclear run-on assays for monitoring RNA-mediated control of transcription (95).

Other laboratories have also observed transcriptional silencing by small RNAs that are complementary to gene promoters. Similar to the targeting strategy for *PR*, Catapano and colleagues designed RNAs to be complementary to sequences at the transcription start site for *Myc* (96). They observed decreased expression of RNA and protein. Formation of the pre-initiation complex for transcription was reduced including decreased recruitment of RNA polymerase II and TFIIB. Other studies, like the original discovery by Morris and co-workers, identified silencing RNAs with complementarity to sequences further upstream from the +1 transcription start site (69,97–100). Promoter-targeted RNAs have also been applied to silencing expression of HIV transcription (101,102). Recently, Tapscott and colleagues have reported silencing of D4Z4 macrosatellite repeats by duplex RNAs targeting the promoter region, providing a potential starting point for treating facioscapulohumeral muscular dystrophy (FSHD) (103).

RNA-mediated gene activation has the potential to add a new dimension to the development of nucleic acid therapeutics because typical duplex RNAs or antisense oligonucleotides are restricted to gene silencing. While their mechanisms of action require further investigation, duplex RNAs have been employed in murine studies to control myocardial infarction size (104) and inhibit liver carcinogenesis (105).

### RNA-mediated transcriptional activation

Some protein transcription factors are known to be associated with gene inhibition in one context and gene activation in another (106–114). Li and colleagues recognized the potential parallel between protein and RNA-mediated control of transcription and tested the ability of promoter-targeted RNAs to activate gene expression (115,116). This study tested the ability of small RNAs to activate three different genes, *E-cadherin, p21* and *VEGF* (115). Small RNAs were targeted to the promoters of each of these genes and upregulation was observed in multiple cell lines at both the RNA and protein levels.

The Li laboratory went on to identify small RNAs that activated other genes including *p21, p53, PAR4, WT1, KLF4, OCT4* and *NKX3* gene transcription and also observed that RNA-mediated gene activation was conserved across mammalian species (117–121). Morris *et al*. also demonstrated RNA-mediated activation of *p21* expression with a requirement for AGO1 expression (98).

As noted above, we had previously observed transcriptional silencing of progesterone receptor (*PR*) expression in human mammary ductal carcinoma T47D cells, a cell line with high basal expression of *PR*. To further test the hypothesis that RNA might activate expression in one context while inhibiting in another we evaluated promoter-targeted RNAs in MCF7 cells that have a low basal level of *PR* expression. We identified several duplex RNAs capable of activating gene transcription (122). Activation of *PR* was also achieved by RNAs that have been chemically modified to reduce off-target effects and improve *in vivo* properties (123). We subsequently observed RNA-mediated activation of cyclooxygenase 2 (*COX-2*) (124) and LDL receptor (125).

Whenever nucleic acids are introduced into cells, there will be a potential for off-target effects (126). Activating or inhibitory promoter-targeted RNAs are also subject to the possibility that observed phenotypes are due to interactions other than those at the intended gene target and examples of these have been reported (127,128). It is important that experimental characterization of target interactions be thorough and that appropriate controls be performed (124) to build a case for on-target effects. As in any field, papers reporting transcriptional silencing and activation vary in the quality and amount of supporting data. Hallmarks of more convincing papers include: (i) use of multiple control oligonucleotides, (ii) application of multiple complementary technologies to bolster central conclusions, and iii) the appearance of follow-up publications that provide more insight into mechanism.

The advent of CRISPR/CAS9-mediated technology has facilitated sequence-specific modification of genomes and has the potential to provide further rigor for analyses. For example, mutation of a seed sequence would be expected to block transcriptional activation or silencing. While CRISPR/CAS9 is an inviting technology, the use of mismatched RNA duplexes remains a quicker and less expensive alternative. In addition, some cell lines where effects have been observed contain multiple chromosomes, making them poor targets for efficient CRISPR-mediated mutation. For targets near transcription start sites, mutations might affect basal expression and obscure interpretation. Regardless of the potential obstacles, well-designed use of CRISPR-CAS9 to validate RNA-mediated transcriptional control would be an important step forward.

### RNAi factors are involved in transcriptional silencing and activation

The RNAs used to achieve transcriptional silencing or activation are duplex RNAs that are identical in structure to those used for standard post-transcriptional RNA interference (7). Because the RNA triggers for transcriptional silencing, transcriptional activation, and post-transcriptional silencing are similar, it is reasonable to hypothesize that RNA-mediated modulation of transcription would involve RNAi factors. The presence of RNAi factors in cell nuclei provides further support for this hypothesis.

Several laboratories have observed that silencing expression of AGO2 reversed transcriptional silencing and transcriptional activation (54,103,122,125,129–131). AGO1 has also been observed to be critical for transcriptional silencing (98,132,133). Interestingly, AGO1 has been reported to directly interact with RNA polymerase II, suggesting a direct physical link between RNAi factors and the core transcription complex (134). Kang and co-workers also observed that expression of SETDB1, a H3-lysine 9 (H3K9) methyl-transferase, was necessary for RNA-mediated silencing of androgen receptor expression (94).

Like AGO, TNRC6A has often been assumed to be localized to cell cytoplasm although it possesses a nuclear localization signal and can be visualized in cell nuclei by microscopy (77). TNRC6A has two paralogs in human cells, TNRC6B and TNRC6C. Silencing all three paralogs reversed transcriptional activation of *COX-2* (124). This finding suggests that TNRC6 proteins are also important factors for RNA-mediated modulation of transcription.

### Nascent RNA transcripts and chromosomal DNA are potential targets for modulatory RNAs

Unlike the duplex RNAs involved in post-transcriptional silencing, the RNAs involved in modulating transcription are not complementary to mRNA. How can duplex RNAs and RNAi factors affect gene expression if they cannot recognize mRNA?

One possibility would be that the duplex RNAs bind directly to chromosomal DNA. This binding could be through triple helix formation (135) or Watson–Crick base pairing (136). Zhang and colleagues have reported that cellular microRNAs can associate with RNA polymerase II and TATA-binding protein (TBP) and bind to TATA box motifs at gene promoters (137). The attraction of this hypothesis is its simplicity—RNA that binds directly to DNA would have an obvious potential to affect binding of transcriptional regulators and chromatin formation.

For RNA–DNA recognition to occur, RNAi factors that are well known to promote binding of RNA to single-stranded RNA would be required to also promote binding to DNA sequences within double-stranded chromosomes that might also be associated with histones. Over the past decades RNA has been repeatedly proven to possess surprising properties that run counter to dogma, but given the chemical and structural differences between single-stranded RNA and duplex DNA, compelling experimental evidence will be needed for RNAi-promoted recognition of chromosomal DNA.

An alternative explanation for gene-specific recognition is that the RNA:RNAi factor complex uses Watson–Crick base-pairing to recognize nascent transcripts that exist in proximity to gene promoters. Large-scale studies of cellular transcription have revealed that much of the genome is transcribed and that transcripts that overlap the 3′ or 5′ termini of genes are common (138–141). These overlapping transcripts provide potential RNA targets for recognition that go beyond mRNA and may be part of a wider web of regulatory RNAs that play previously unsuspected roles in epigenetic regulation of gene expression (142).

Several overlapping promoter transcripts have been implicated in RNA-mediated activation or silencing. The *PR* (143), *COX-2* (124) and *LDLR* (125) promoters all express RNAs that overlap their +1 transcription start sites and are complementary to duplex RNAs that modulate transcription. Catapano and colleagues identified a transcript overlapping the *Myc* promoter that was also implicated in targeting RNA-mediated transcriptional silencing (96). Promoter-associated RNAs have also been implicated in the ability of small RNAs to modulate expression of ubiquitin C (98), androgen receptor (94) and a transduced model gene expression green fluorescent protein (131).

Several lines of evidence support involvement of promoter RNAs in transcriptional regulation (121,144). Upon addition of duplex RNA complementary to gene promoters, RNA immunoprecipitation using anti-AGO2 antibody revealed that AGO2 is recruited to promoter transcripts. Antisense oligonucleotides are powerful tools for modulating the action of promoter RNAs and other noncoding RNA species (145) and addition of an antisense oligonucleotide designed to induce RNase H-mediated cleavage of the promoter transcript causes reversal of RNA-mediated gene activation. Taken together, these data suggest that addition of a duplex promoter-targeted RNA to cells triggers formation of a ribonucleoprotein complex between the promoter transcript, small RNA, AGO2, TNRC6 and possibly other factors.

Upon binding, AGO2 has the potential to cleave target transcripts and it is possible that cleavage of a nascent transcript might directly trigger transcriptional change. To test this hypothesis we compared a fully complementary duplex RNA targeting the *COX-2* promoter with a duplex RNA with central mismatches relative to the target site. Central mismatches disrupt the ability of AGO2 to cleave substrate without affecting its ability to bind target sequences (146). Gene activation by the mismatch-containing duplex RNA was as effective as activation by the fully complementary RNA (124). This finding suggests that binding to nascent transcripts is a potential trigger for activation, at least at the *COX-2* locus, and not cleavage of the transcript.

RNA and RNAi factors have an enormous potential for regulation. It is important to consider the possibility that RNAi-mediated cleavage of nascent transcripts might trigger modulation of gene expression. However, to date, there is no strong evidence for this mechanism.

### Modulation occurs in *cis* relative to the target gene locus

**Figure 4.**
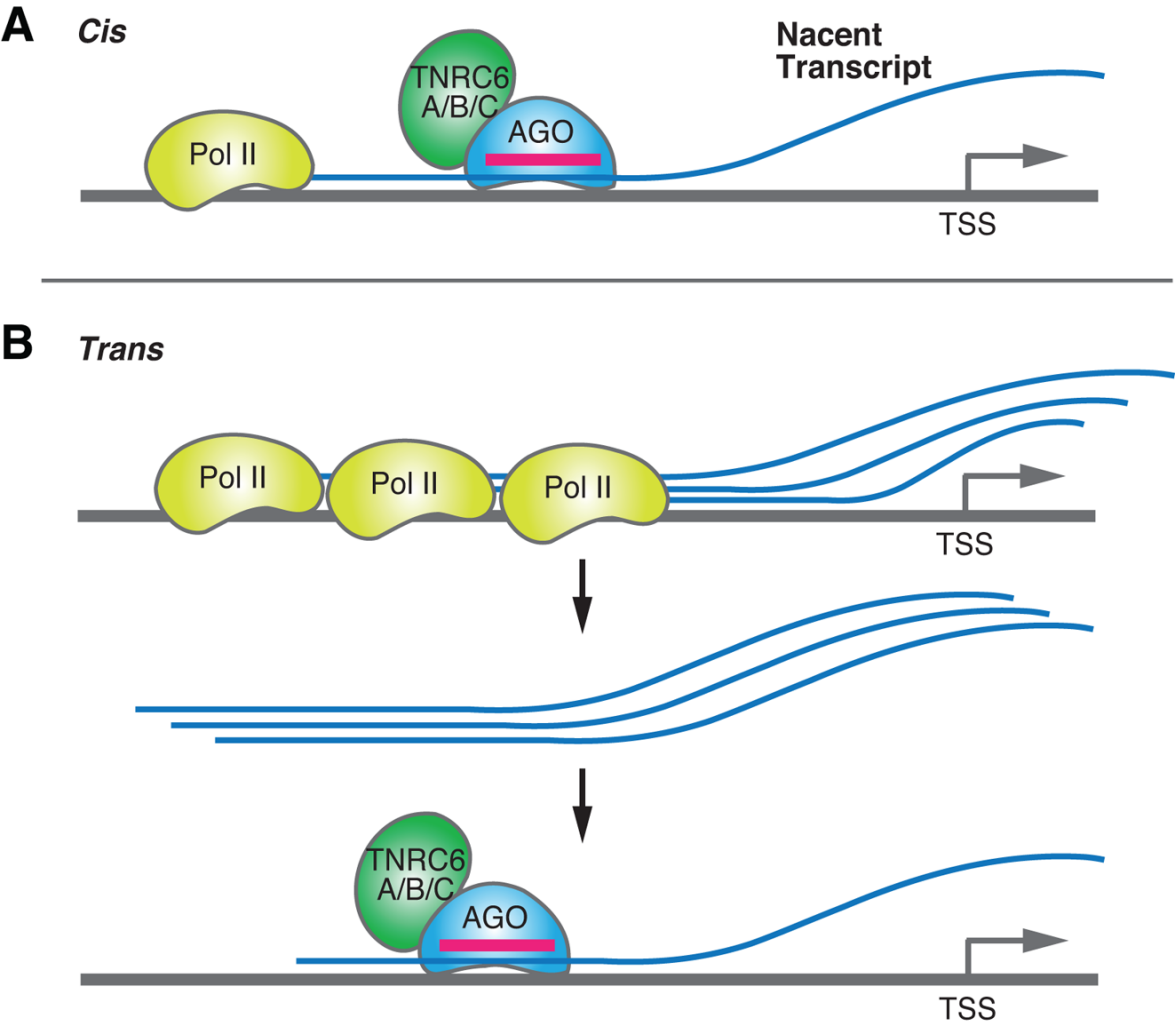
*Cis* and *trans* acting nascent RNAs. (**A**) A low copy number per cell of nascent RNA increases the likelihood of the RNA acting in *cis*. The RNA remains bound to the site of transcription. (**B**) A high copy number per cell of nascent RNA increases the likelihood that the RNAs will act in *trans*. The nascent RNAs act at a distance from their site of transcription and bind to other sites along the genome.

Nascent RNA transcripts may act at the site where they are transcribed (in *cis*) or they may act at a distant site (in *trans*). Whether an RNA acts in *cis* or *trans* relative to its target gene is one determinant of its mechanism of action and careful characterization is necessary to achieve persuasive results (147). For example, the quantity of an RNA transcript in an individual cell is rarely measured but can have critical implications for an underlying mechanism of action. The sensitivity of PCR techniques allows for identification of RNA transcripts spanning a wide range of expression levels. This data can be interpreted in a number of ways. An RNA that is expressed at only one copy per hundred cells might be the product of background transcription. An RNA present at just a few copies per cells would be relatively more likely to function in *cis* rather than in *trans* (Figure 4A), whereas a highly expressed transcript present in hundreds to thousands of copies per cell would be more likely to function in *trans* (Figure 4B). For the *COX-2* locus, the levels of promoter RNA involved in transcriptional modulation were measured on a per cell basis. This RNA transcript was detected at one or two copies per cell (148). This low number suggests that the RNA is involved in *cis* relative to the target gene rather than in *trans*.

Rossi, Burnett *et al*. have noted a similar role for nascent transcripts in the mechanism of action for RNA-mediated activation (131,137). They observed that RNA-mediated activation of a reporter gene under control of a cytomegalovirus promoter was dependent on AGO2 and that nascent transcripts at the promoter were the direct targets. Cleavage of the nascent transcript was not necessary, leading to the conclusion that the transcript was a scaffold and that activation occurred in *cis* relative to the target gene. Similarly, Giles and coworkers have shown that AGO2 binds to nascent tRNA and can also block gene transcription in *cis* (149).

Interestingly, an in *cis* mechanism for mammalian RNA-mediated modulation of transcription is quite similar to that originally proposed by Grewal and Moazed for RNA-mediated control of transcription in *S. pombe* (150). The commonality of mechanisms suggests an evolutionarily conserved pathway for RNA-mediated control of transcription.

## LESSONS FROM PROTEIN TRANSCRIPTION FACTORS

### Mechanisms of transcriptional inhibition

The studies describing RNA-mediated modulation of gene expression have begun to define a mechanism of action. Promoter-targeted RNAs are loaded onto AGO2 protein in the cytoplasm and the RNA:AGO2 complex subsequently enters the nucleus. Once in the nucleus, the guide RNA hybridizes by Watson–Crick base-paring to a complementary sequence within a nascent transcript at the gene promoter. RNA immunoprecipitation indicates that AGO2 and TNRC6 proteins are also associated with the nascent transcript.

The guide strand RNA:AGO2:TNRC6 complex near the promoter region could potentially affect the binding of transcription factors, alter covalent modifications of nucleosomal histones and/or DNA bases, change nucleosome positioning, enhance or repress recruitment of RNA polymerase II, and increase or decrease transcription of mRNA (151–153).

**Figure 5.**
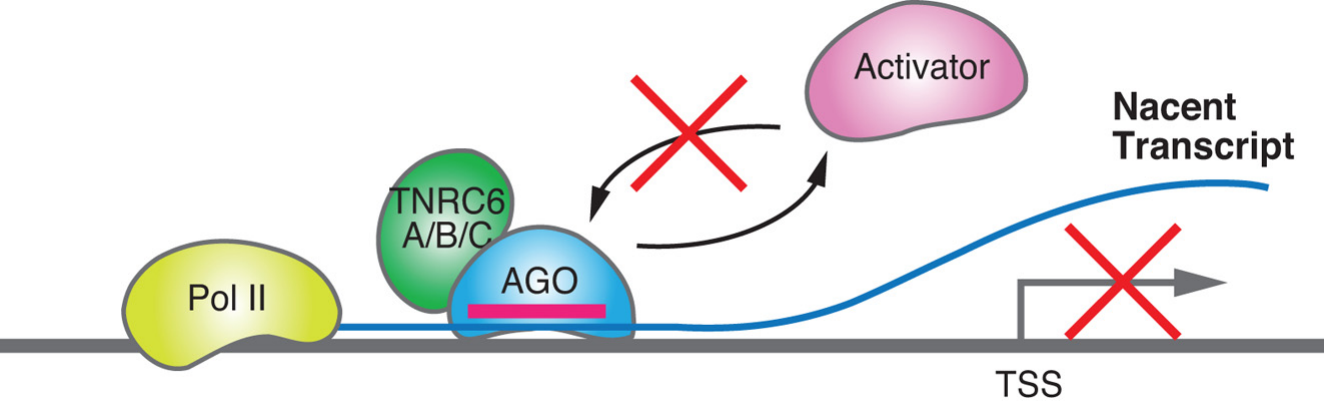
Scheme showing one potential mechanism for repression. The RNA:AGO2:TRNC6 complex binds at a promoter and disrupts the assembly of proteins necessary for gene activation.

How can binding of an RNA:AGO2:TNRC6 complex at a promoter lead to altered transcription of mRNA? The mechanism for inhibiting gene expression may be relatively straightforward. Activation of gene expression requires that accessory proteins act in concert to recruit RNA polymerase II and trigger transcription. By changing the factor occupancy at an activated promoter, critical protein:protein interactions needed for gene activation are blocked (154–159). When an RNA:RNAi factor complex binds to a nascent transcript it can act as a repressor by blocking activating factors or displacing factors that are already present on the promoter (Figure 5).

### Mechanisms of transcriptional activation

For gene activation, the RNA:AGO2:TNRC6 complex may mimic the action of protein transcription factors. Promoter-targeted RNAs modulate the expression of genes such as *COX-2* (124), *PR* (143), and LDL receptor (125). These genes share a common feature - they have a low basal level of expression that can be activated to a higher level. The promoters for these genes are already occupied by components of the transcription machinery that are poised to respond to environmental signals that trigger changes in gene expression.

**Figure 6.**
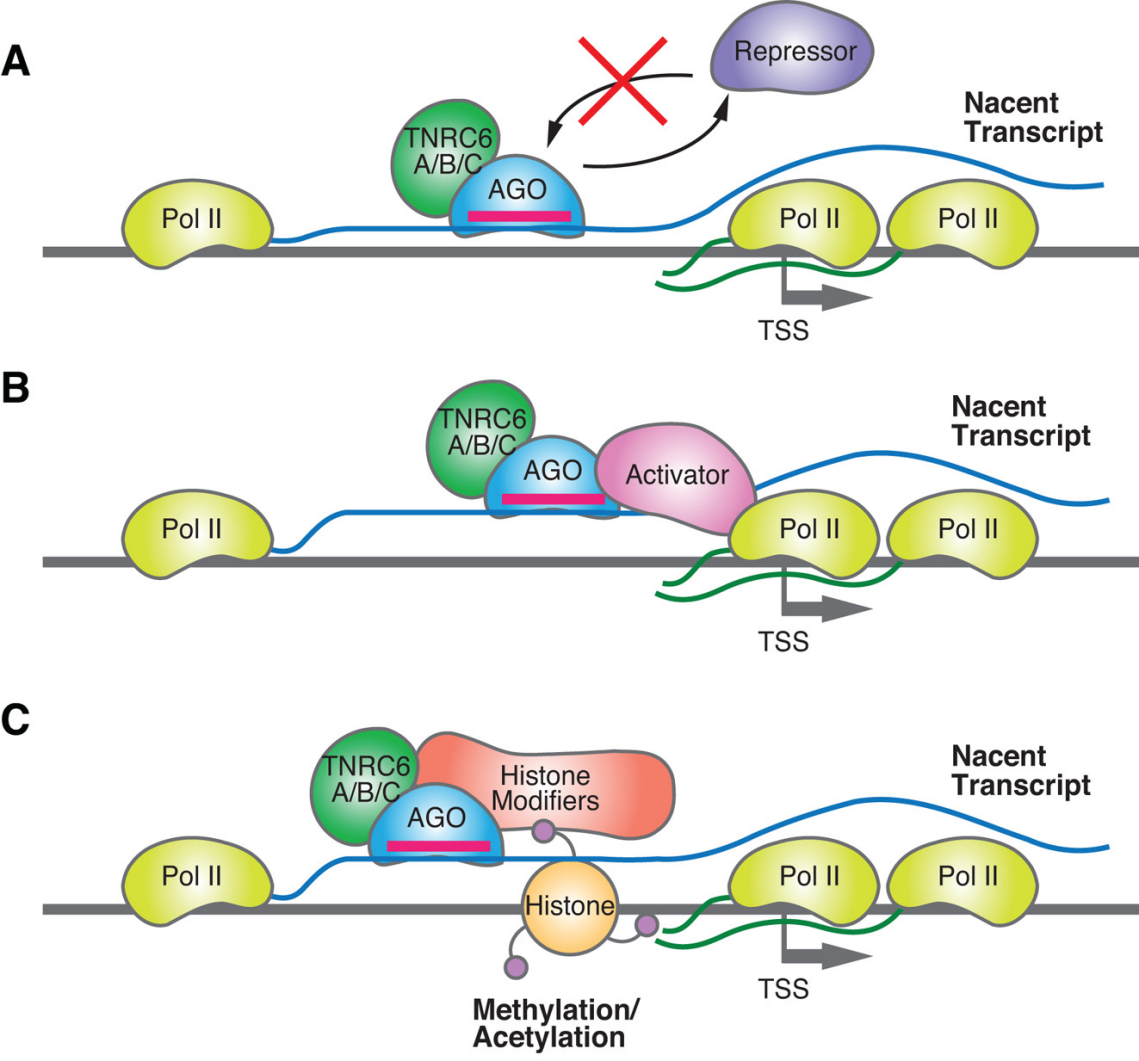
Scheme showing potential mechanisms for RNA-mediated gene activation that are based on known mechanisms for activation by protein transcription factors. (**A**) Activation by blocking the binding of one or more proteins needed for repression. (**B**) Activation by promoting the binding of an activating factor. (**C**) Activation by inducing histone modifications.

Transcription factors can activate gene expression through several mechanisms and similar mechanisms might also be adopted by RNAi factors. The binding of factors inhibiting transcription can be sterically blocked by prior or competing binding of proteins to a region overlapping or proximal to the repressor-binding site (106,160–162) (Figure 6A). Alternatively, the binding of transcription factors can cooperatively enhance the association of other factors to an adjacent region (163–165) (Figure 6B). Moreover, transcription factors can adopt altered conformations upon ligand binding, strengthening its association with a coactivator or weakening an association with a corepressor (166,167).

In the context of chromatin, transcription factors can change the translational and/or rotational phasing of nucleosomes relative to the transcription start site, thereby increasing the access of transcription factors to their chromatin target sequences (168). Covalent modifications of nucleosomal histones and DNA surrounding the transcription start site can be regulated by binding of factors that affect the access or action of a chromatin-modifying enzyme to a chromatin-regulated promoter (165,169) (Figure 6C).

Binding of RNA:AGO2:TNRC6 complexes to nascent transcripts has the potential to activate gene expression by any of the mechanisms noted above (Figure 6). RNAi factor complexes are not canonical protein transcription factors, but it is important to note that transcriptional activation does not require highly evolved endogenous proteins. Activation domains can be peptides, small RNAs, or small molecules (170). The wide range of domain types suggests that the orientation or composition of a domain at a promoter is not as essential as the basal expression level and its ability to respond to environmental stimuli. Similar mechanisms may be broadly applicable to enhancer RNAs and other mechanisms for RNA-mediated activation (171).

## miRNAs IN CELL NUCLEI

In the cytoplasm, miRNAs interact with RNAi factors to recognize sequences within the 3′-untranslated region of mRNA and control protein translation. miRNAs, however, have also been detected in the nuclei of mammalian cells after genome-wide sequencing studies or large-scale PCR (82,172–174). One miRNA, miRNA-29b, appears to be predominantly localized to the nucleus and contains a hexanucleotide that is responsible for nuclear import (175). Recent data has suggested the possibility for miRNAs to play a role in splicing and other noncanonical functions in nuclei (176). Small RNA binding sites are frequently observed at gene promoters and ChIP experiments have revealed AGO bound near promoter sites (177,178).

### Modulating transcription with miRNAs

miRNA-induced transcriptional regulation has been reported in mammalian systems, supporting the potential for endogenous control. The first study found that miRNA-373 could upregulate *E-cadherin* and *CSDC2*, both of which contained complimentary sequences in their promoters (179). Another study found that a miRNA encoded within the promoter of the gene *POLR3D* could act in *cis* to recruit AGO1 and other factors to silence *POLR3D* expression (180). More recently, Huang et al. found that depletion of miRNA-774 caused a down-regulation of Ccnb1 (181). Our laboratory identified miRNAs that inhibited the expression of *PR* (182) through a mechanism involving AGO2 and H3K9 methylation. At the *COX-2* locus, we identified miRNA-589 as having two complementary sites at the *COX-2* promoter and implicated it in the activation of COX-2 expression (124).

## RNAi-MEDIATED MODULATION OF ALTERNATIVE SPLICING

Alternative splicing is a nuclear process that leads to the production of diverse protein isoforms or modulates the stability of proteins. These variants have different functions and the regulation of isoform production is necessary for cell function, growth, and development. Splicing is controlled by interactions between pre-mRNA and protein splicing factors. The combination of duplex RNAs and nuclear RNAi factors have the potential to disrupt these interactions and provide another layer of regulation.

### Modulating splicing by affecting histone modification

**Figure 7.**
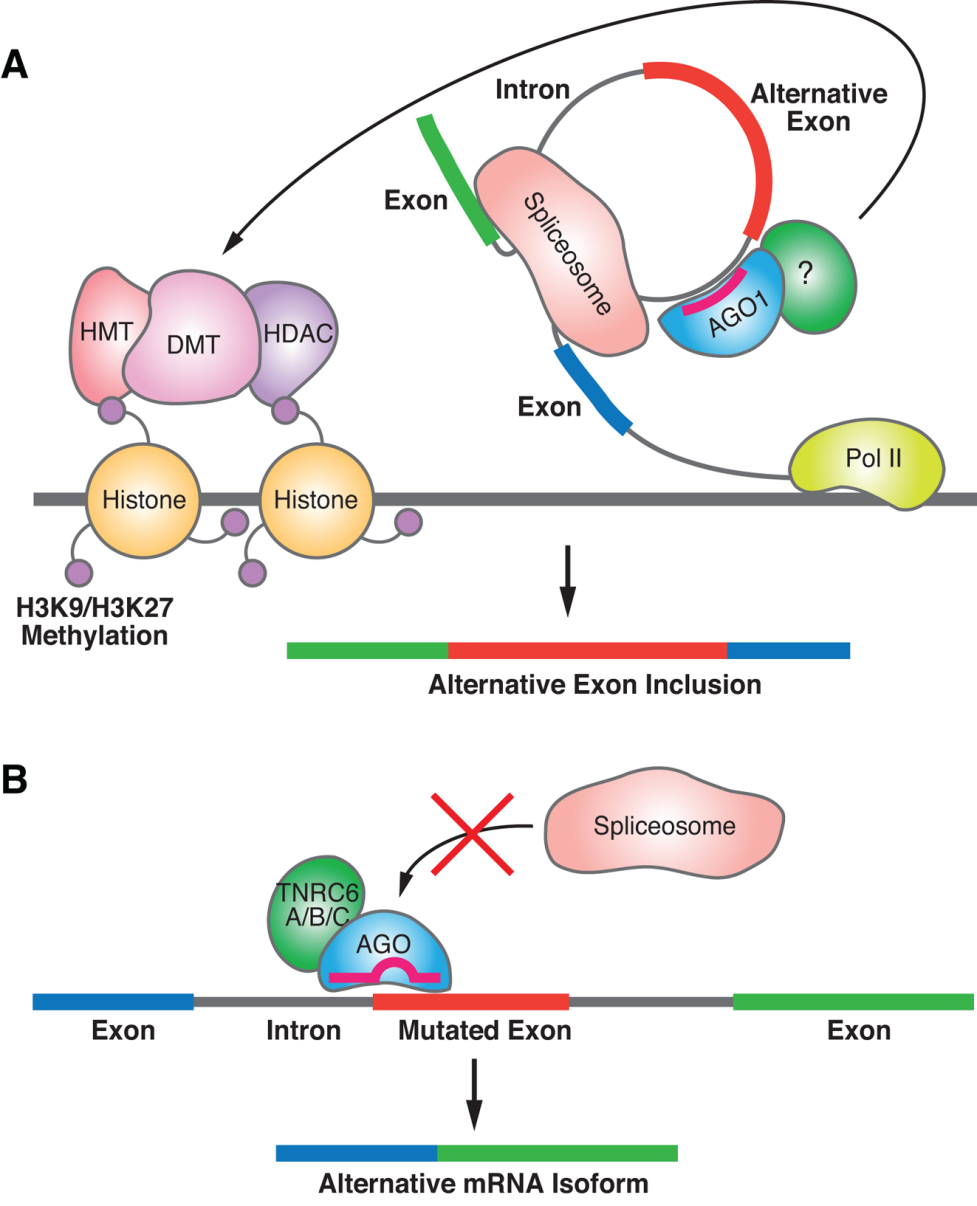
Scheme showing potential mechanism for RNA-mediated control of alternative splicing. (**A**) RNA-mediated binding of AGO1 alters histone modifications, affects the rate of transcription, and alters alternative splicing. (**B**) Binding of an RNA:RNAi factor complex near a splice site blocks association of the spliceosome and redirects alternative splicing.

Splicing can be coupled with transcription, and the kinetics of RNA polymerase elongation has the potential to affect recruitment of splicing factors (183). Kornblihtt *et al*. reported that duplex RNAs targeting intronic or exonic sequences near alternative exons can affect splicing of the exon (184). RNAi-associated alternative splicing required expression of AGO1 and was associated with increase in histone marks Lys9 dimethylation and Lys27 trimethylation at histone H3. AGO1 was subsequently reported to be associated with transcriptional enhancers and noncoding enhancer RNAs that may also affect splicing (185). Taken together, these results suggest that binding of small RNA/AGO complexes in proximity to promoters can affect the kinetics of transcriptional elongation, thereby shifting the outcome of alternative splicing among isoforms (Figure 7A).

AGO1 and AGO2 have also been reported to couple chromatin silencing to alternative splicing, although the role of guide RNA in this process, if any, is unclear (186). Data suggest that antisense transcripts, rather than just mRNA or pre-mRNA, may be major targets for splice-altering duplex RNAs that affect histone modification (187). Most recently, chromatin immunoprecipitation coupled to high throughput DNA sequencing (ChIP-Seq) has been used to correlate protein binding with splicing and results suggest that AGO1 may participate with HP1α, CTCF and other proteins in programming a chromatin code to supply a layer of control for alternative splicing (188).

### Modulating splicing by blocking sites near exon/intron junctions

Several diseases can be at least partially alleviated by modulating splicing to yield mRNAs that produce protein isoforms that are beneficial to patients. For example Duchenne muscular dystrophy (DMD) is caused by a truncation in dystrophin protein due to a premature stop codon (189). Antisense oligonucleotides that target sequences near an intron/exon junction cause the exon to be skipped, correcting the reading frame and allowing expression of a longer and partially functional protein (190). Oligomers that alter splicing for dystrophin and SMN2 protein (the molecular origin of spinal muscular atrophy) are currently being tested in separate clinical trials (191,192).

Antisense oligonucleotides act by binding pre-mRNA and blocking association of splicing factors (193). Our laboratory tested the hypothesis that duplex RNAs in concert with nuclear RNAi factors would bind to exon-intron junctions and produce similar effects on splicing (130) (Figure 7B). We first analyzed the effect of duplex RNA on a classical model of splicing consisting of an engineered luciferase gene containing a β-globin intron (194) and observed altered splicing and increased expression of active luciferase (195). We subsequently observed that duplex RNAs could also modulate splicing of dystrophin and SMN2. Single-stranded silencing RNAs (ss-siRNAs) are chemically modified single-stranded oligomers that can act through the RNAi pathway and silence gene expression in cells and animals (13,196). ss-siRNAs are also able to modulate splicing (197). Modulation of splicing by both ss-siRNAs and duplex RNAs is dependent on expression of AGO2.

RNAi-regulated splicing of endogenous genes has not been reported to date, but RNA sequencing of AGO2-bound species in human cell nuclei has revealed many potential binding sites near exon/intron boundaries (176), suggesting that nuclear AGO may have a role in alternative splicing. Drosophila AGO2 has also been identified as being enriched near splice sites for genes where splicing is changed by knockdown of AGO2 (198). As with mammalian AGO2, the full implications for regulation of splicing remain unclear.

## CONCLUSIONS

The mammalian nucleus contains many different species of RNA, each of which must be regulated and many of which have the potential to be regulators. Experimental data have built a strong case for the existence of RNAi factors in mammalian somatic cell nuclei, consistent with their presence in yeast, worms, and plants. These nuclear RNA factors can act in conjunction with small RNAs to regulate transcription and splicing. Data suggest that multiple mechanisms are involved and that these mechanisms share considerable similarity to mechanisms of protein transcription or splicing factors. The distinguishing feature relative to protein factors is that the small RNA provides a programmable specificity module that can be more readily adapted for recognition of many different RNA species.

This programmable specificity of nuclear RNA:RNAi factor complexes has the potential to confer a significant evolutionary advantage and may represent a new area for miRNA function. Experiments using model endogenous genes have demonstrated that miRNAs can regulate gene transcription, but the broad importance of miRNAs in cell nuclei is not yet clear. Gaining a better appreciation for the impact of nuclear RNAi on mammalian physiology and a more detailed mechanistic understanding will be primary goals for future research.

## References

[1] Zhou H.L., Luo G., Wise J.A., Lou H. (2014). Regulation of alternative splicing by local histone modifications: potential roles for RNA-guided mechanisms. Nucleic Acids Res..

[2] Braunschweig U., Gueroussov S., Plocik A.M., Graveley B.R., Blencowe B.J. (2013). Dynamic integration of splicing within gene regulatory pathways. Cell.

[3] Kornblihtt A.R., Schor I.E., Allo M., Dujardin G., Petrillo E., Munoz M.J. (2013). Alternative splicing: a pivotal step between eukaryotic transcription and translation. Nat. Rev. Mol. Cell. Biol..

[4] Ipsaro J.J., Joshua-Tor L. (2015). From guide to target: molecular insights into eukaryotic RNA-interference machinery. Nat. Struct. Mol. Biol..

[5] Wilson R.C., Doudna J.A. (2013). Molecular mechanisms of RNA interference. Annu. Rev. Biophys..

[6] Stark A., Brennecke J., Bushati N., Russell R.B., Cohen S.M. (2005). Animal MicroRNAs confer robustness to gene expression and have a significant impact on 3′UTR evolution. Cell.

[7] Elbashir S.M., Harborth J., Lendeckel W., Yalcin A., Weber K., Tuschl T. (2001). Duplexes of 21-nucleotide RNAs mediate RNA interference in cultured mammalian cells. Nature.

[8] Meister G., Landthaler M., Patkaniowska A., Dorsett Y., Teng G., Tuschl T. (2004). Human Argonaute2 mediates RNA cleavage targeted by miRNAs and siRNAs. Mol. Cell.

[9] Rand T.A., Ginalski K., Grishin N.V., Wang X. (2004). Biochemical identification of Argonaute 2 as the sole protein required for RNA-induced silencing complex activity. Proc. Natl. Acad. Sci. U.S.A..

[10] Liu J., Carmell M.A., Rivas F.V., Marsden C.G., Thomson J.M., Song J.J., Hammond S.M., Joshua-Tor L., Hannon G.J. (2004). Argonaute2 is the catalytic engine of mammalian RNAi. Science.

[11] Jakymiw A., Lian S., Eystathioy T., Li S., Satoh M., Hamel J.C., Fritzler M.J., Chan E.K. (2005). Disruption of GW bodies impairs mammalian RNA interference. Nat. Cell Biol..

[12] Eulalio A., Huntzinger E., Izaurralde E. (2008). GW182 interaction with Argonaute is essential for miRNA-mediated translational repression and mRNA decay. Nat. Struct. Mol. Biol..

[13] Lima W.F., Prakash T.P., Murray H.M., Kinberger G.A., Li W., Chappell A.E., Li C.S., Murray S.F., Gaus H., Seth P.P. (2012). Single-stranded siRNAs activate RNAi in animals. Cell.

[14] Ha M., Kim V.N. (2014). Regulation of microRNA biogenesis. Nat. Rev. Mol. Cell Biol..

[15] Hammond S.M. (2015). An overview of microRNAs. Adv. Drug Deliv. Rev..

[16] Jonas S., Izaurralde E. (2015). Towards a molecular understanding of microRNA-mediated gene silencing. Nat. Rev. Genet..

[17] Meister G. (2013). Argonaute proteins: functional insights and emerging roles. Nat. Rev. Genet..

[18] Ruda V.M., Chandwani R., Sehgal A., Bogorad R.L., Akinc A., Charisse K., Tarakhovsky A., Novobrantseva T.I., Koteliansky V. (2014). The roles of individual mammalian argonautes in RNA interference in vivo. PLoS One.

[19] Liu J., Rivas F.V., Wohlschlegel J., Yates J.R. 3rd, Parker R., Hannon G.J. (2005). A role for the P-body component GW182 in microRNA function. Nat. Cell Biol..

[20] Meister G., Landthaler M., Peters L., Chen P.Y., Urlaub H., Luhrmann R., Tuschl T. (2005). Identification of novel argonaute-associated proteins. Curr. Biol..

[21] Pfaff J., Meister G. (2013). Argonaute and GW182 proteins: an effective alliance in gene silencing. Biochem. Soc. Trans..

[22] Ui-Tei K., Nishi K., Takahashi T., Nagasawa T. (2012). Thermodynamic Control of Small RNA-Mediated Gene Silencing. Front. Genet..

[23] Martinez J., Patkaniowska A., Urlaub H., Luhrmann R., Tuschl T. (2002). Single-stranded antisense siRNAs guide target RNA cleavage in RNAi. Cell.

[24] Schirle N.T., MacRae I.J. (2012). The crystal structure of human Argonaute2. Science.

[25] Elkayam E., Kuhn C.D., Tocilj A., Haase A.D., Greene E.M., Hannon G.J., Joshua-Tor L. (2012). The structure of human argonaute-2 in complex with miR-20a. Cell.

[26] Salomon W.E., Jolly S.M., Moore M.J., Zamore P.D., Serebrov V. (2015). Single-molecule imaging reveals that argonaute reshapes the binding properties of its nucleic acid guides. Cell.

[27] Holoch D., Moazed D. (2015). RNA-mediated epigenetic regulation of gene expression. Nat. Rev. Genet..

[28] Creamer K.M., Partridge J.F. (2011). RITS-connecting transcription, RNA interference, and heterochromatin assembly in fission yeast. Wiley Interdiscip. Rev. RNA.

[29] Rogers K., Chen X. (2013). Biogenesis, turnover, and mode of action of plant microRNAs. Plant Cell.

[30] Castel S.E., Martienssen R.A. (2013). RNA interference in the nucleus: roles for small RNAs in transcription, epigenetics and beyond. Nat. Rev. Genet..

[31] Cecere G., Grishok A. (2014). A nuclear perspective on RNAi pathways in metazoans. Biochim. Biophys. Acta.

[32] Wassenegger M., Heimes S., Riedel L., Sanger H.L. (1994). RNA-directed de novo methylation of genomic sequences in plants. Cell.

[33] Mette M.F., Aufsatz W., van der Winden J., Matzke M.A., Matzke A.J. (2000). Transcriptional silencing and promoter methylation triggered by double-stranded RNA. EMBO J..

[34] Zilberman D., Cao X., Jacobsen S.E. (2003). ARGONAUTE4 control of locus-specific siRNA accumulation and DNA and histone methylation. Science.

[35] McCue A.D., Panda K., Nuthikattu S., Choudury S.G., Thomas E.N., Slotkin R.K. (2015). ARGONAUTE 6 bridges transposable element mRNA-derived siRNAs to the establishment of DNA methylation. EMBO J..

[36] Underwood C.J., Martienssen R.A. (2015). Argonautes team up to silence transposable elements in Arabidopsis. EMBO J..

[37] Duan C.G., Zhang H., Tang K., Zhu X., Qian W., Hou Y.J., Wang B., Lang Z., Zhao Y., Wang X. (2015). Specific but interdependent functions for Arabidopsis AGO4 and AGO6 in RNA-directed DNA methylation. EMBO J..

[38] Haag J.R., Pikaard C.S. (2011). Multisubunit RNA polymerases IV and V: purveyors of non-coding RNA for plant gene silencing. Nat. Rev. Mol. Cell. Biol..

[39] Wierzbicki A.T., Haag J.R., Pikaard C.S. (2008). Noncoding transcription by RNA polymerase Pol IVb/Pol V mediates transcriptional silencing of overlapping and adjacent genes. Cell.

[40] Zhao Y., Chen X. (2014). Noncoding RNAs and DNA methylation in plants. Natl. Sci. Rev..

[41] Zhu Y., Rowley M.J., Bohmdorfer G., Wierzbicki A.T. (2013). A SWI/SNF chromatin-remodeling complex acts in noncoding RNA-mediated transcriptional silencing. Mol. Cell.

[42] Verdel A., Jia S., Gerber S., Sugiyama T., Gygi S., Grewal S.I., Moazed D. (2004). RNAi-mediated targeting of heterochromatin by the RITS complex. Science.

[43] Martienssen R., Moazed D. (2015). RNAi and heterochromatin assembly. Cold Spring. Harb. Perspect. Biol..

[44] Wu L., Mao L., Qi Y. (2012). Roles of dicer-like and argonaute proteins in TAS-derived small interfering RNA-triggered DNA methylation. Plant Physiol..

[45] Halic M., Moazed D. (2010). Dicer-independent primal RNAs trigger RNAi and heterochromatin formation. Cell.

[46] Reinhart B.J., Bartel D.P. (2002). Small RNAs correspond to centromere heterochromatic repeats. Science.

[47] Volpe T.A., Kidner C., Hall I.M., Teng G., Grewal S.I., Martienssen R.A. (2002). Regulation of heterochromatic silencing and histone H3 lysine-9 methylation by RNAi. Science.

[48] Jia S., Kobayashi R., Grewal S.I. (2005). Ubiquitin ligase component Cul4 associates with Clr4 histone methyltransferase to assemble heterochromatin. Nat. Cell Biol..

[49] Horn P.J., Bastie J.N., Peterson C.L. (2005). A Rik1-associated, cullin-dependent E3 ubiquitin ligase is essential for heterochromatin formation. Genes Dev..

[50] Hong E.J., Villen J., Gerace E.L., Gygi S.P., Moazed D. (2005). A cullin E3 ubiquitin ligase complex associates with Rik1 and the Clr4 histone H3-K9 methyltransferase and is required for RNAi-mediated heterochromatin formation. RNA Biol..

[51] Kowalik K.M., Shimada Y., Flury V., Stadler M.B., Batki J., Buhler M. (2015). The Paf1 complex represses small-RNA-mediated epigenetic gene silencing. Nature.

[52] Guerin T.M., Palladino F., Robert V.J. (2014). Transgenerational functions of small RNA pathways in controlling gene expression in C. elegans. Epigenetics.

[53] Knight S.W., Bass B.L. (2001). A role for the RNase III enzyme DCR-1 in RNA interference and germ line development in Caenorhabditis elegans. Science.

[54] Grishok A., Pasquinelli A.E., Conte D., Li N., Parrish S., Ha I., Baillie D.L., Fire A., Ruvkun G., Mello C.C. (2001). Genes and mechanisms related to RNA interference regulate expression of the small temporal RNAs that control C. elegans developmental timing. Cell.

[55] Ketting R.F., Fischer S.E., Bernstein E., Sijen T., Hannon G.J., Plasterk R.H. (2001). Dicer functions in RNA interference and in synthesis of small RNA involved in developmental timing in C. elegans. Genes Dev..

[56] Gu W., Shirayama M., Conte D., Vasale J., Batista P.J., Claycomb J.M., Moresco J.J., Youngman E.M., Keys J., Stoltz M.J. (2009). Distinct argonaute-mediated 22G-RNA pathways direct genome surveillance in the C. elegans germline. Mol. Cell.

[57] Guang S., Bochner A.F., Pavelec D.M., Burkhart K.B., Harding S., Lachowiec J., Kennedy S. (2008). An Argonaute transports siRNAs from the cytoplasm to the nucleus. Science.

[58] Guang S., Bochner A.F., Burkhart K.B., Burton N., Pavelec D.M., Kennedy S. (2010). Small regulatory RNAs inhibit RNA polymerase II during the elongation phase of transcription. Nature.

[59] Gu S.G., Pak J., Guang S., Maniar J.M., Kennedy S., Fire A. (2012). Amplification of siRNA in Caenorhabditis elegans generates a transgenerational sequence-targeted histone H3 lysine 9 methylation footprint. Nat. Genet..

[60] Burkhart K.B., Guang S., Buckley B.A., Wong L., Bochner A.F., Kennedy S. (2011). A pre-mRNA-associating factor links endogenous siRNAs to chromatin regulation. PLoS Genet..

[61] Burton N.O., Burkhart K.B., Kennedy S. (2011). Nuclear RNAi maintains heritable gene silencing in Caenorhabditis elegans. Proc. Natl. Acad. Sci. U.S.A..

[62] Zeng Y., Cullen B.R. (2002). RNA interference in human cells is restricted to the cytoplasm. RNA.

[63] Vickers T.A., Koo S., Bennett C.F., Crooke S.T., Dean N.M., Baker B.F. (2003). Efficient reduction of target RNAs by small interfering RNA and RNase H-dependent antisense agents. A comparative analysis. J. Biol. Chem..

[64] Stalder L., Heusermann W., Sokol L., Trojer D., Wirz J., Hean J., Fritzsche A., Aeschimann F., Pfanzagl V., Basselet P. (2013). The rough endoplasmatic reticulum is a central nucleation site of siRNA-mediated RNA silencing. EMBO J..

[65] Ikeda K., Satoh M., Pauley K.M., Fritzler M.J., Reeves W.H., Chan E.K. (2006). Detection of the argonaute protein Ago2 and microRNAs in the RNA induced silencing complex (RISC) using a monoclonal antibody. J. Immunol. Methods.

[66] Sen G.L., Blau H.M. (2005). Argonaute 2/RISC resides in sites of mammalian mRNA decay known as cytoplasmic bodies. Nat. Cell Biol..

[67] Morris K.V., Chan S.W., Jacobsen S.E., Looney D.J. (2004). Small interfering RNA-induced transcriptional gene silencing in human cells. Science.

[68] Kawasaki H., Taira K. (2004). Induction of DNA methylation and gene silencing by short interfering RNAs in human cells. Nature.

[69] Ting A.H., Schuebel K.E., Herman J.G., Baylin S.B. (2005). Short double-stranded RNA induces transcriptional gene silencing in human cancer cells in the absence of DNA methylation. Nat. Genet..

[70] Taira K. (2006). Induction of DNA methylation and gene silencing by short interfering RNAs in human cells. Nature.

[71] Robb G.B., Brown K.M., Khurana J., Rana T.M. (2005). Specific and potent RNAi in the nucleus of human cells. Nat. Struct. Mol. Biol..

[72] Ohrt T., Mutze J., Staroske W., Weinmann L., Hock J., Crell K., Meister G., Schwille P. (2008). Fluorescence correlation spectroscopy and fluorescence cross-correlation spectroscopy reveal the cytoplasmic origination of loaded nuclear RISC in vivo in human cells. Nucleic Acids Res..

[73] Rudel S., Flatley A., Weinmann L., Kremmer E., Meister G. (2008). A multifunctional human Argonaute2-specific monoclonal antibody. RNA.

[74] Wei Y., Li L., Wang D., Zhang C.Y., Zen K. (2014). Importin 8 regulates the transport of mature microRNAs into the cell nucleus. J. Biol. Chem..

[75] Weinmann L., Hock J., Ivacevic T., Ohrt T., Mutze J., Schwille P., Kremmer E., Benes V., Urlaub H., Meister G. (2009). Importin 8 is a gene silencing factor that targets argonaute proteins to distinct mRNAs. Cell.

[76] Till S., Lejeune E., Thermann R., Bortfeld M., Hothorn M., Enderle D., Heinrich C., Hentze M.W., Ladurner A.G. (2007). A conserved motif in Argonaute-interacting proteins mediates functional interactions through the Argonaute PIWI domain. Nat. Struct. Mol. Biol..

[77] Nishi K., Nishi A., Nagasawa T., Ui-Tei K. (2013). Human TNRC6A is an Argonaute-navigator protein for microRNA-mediated gene silencing in the nucleus. RNA.

[78] Schraivogel D., Schindler S.G., Danner J., Kremmer E., Pfaff J., Hannus S., Depping R., Meister G. (2015). Importin-beta facilitates nuclear import of human GW proteins and balances cytoplasmic gene silencing protein levels. Nucleic Acids Res..

[79] Pfaff J., Hennig J., Herzog F., Aebersold R., Sattler M., Niessing D., Meister G. (2013). Structural features of Argonaute-GW182 protein interactions. Proc. Natl. Acad. Sci. U.S.A..

[80] White E., Schlackow M., Kamieniarz-Gdula K., Proudfoot N.J., Gullerova M. (2014). Human nuclear Dicer restricts the deleterious accumulation of endogenous double-stranded RNA. Nat. Struct. Mol. Biol..

[81] Hirsch M., Helm M. (2015). Live cell imaging of duplex siRNA intracellular trafficking. Nucleic Acids Res..

[82] Gagnon K.T., Li L., Chu Y., Janowski B.A., Corey D.R. (2014). RNAi factors are present and active in human cell nuclei. Cell Rep..

[83] Gagnon K.T., Li L., Janowski B.A., Corey D.R. (2014). Analysis of nuclear RNA interference in human cells by subcellular fractionation and Argonaute loading. Nat. Protoc..

[84] Matsui M., Li L., Janowski B.A., Corey D.R. (2015). Reduced expression of argonaute 1, argonaute 2, and TRBP changes levels and intracellular distribution of RNAi factors. Sci. Rep..

[85] Hauptmann J., Schraivogel D., Bruckmann A., Manickavel S., Jakob L., Eichner N., Pfaff J., Urban M., Sprunck S., Hafner M. (2015). Biochemical isolation of Argonaute protein complexes by Ago-APP. Proc. Natl. Acad. Sci. U.S.A..

[86] Behlke K.A.L.M.A. (2015). Cellular localization affects knockdown efficiency of long non-coding RNAs. Nucleic Acids Res..

[87] Gao M., Wei W., Li M.M., Wu Y.S., Ba Z., Jin K.X., Liao Y.Q., Adhikari S., Chong Z., Zhang T. (2014). Ago2 facilitates Rad51 recruitment and DNA double-strand break repair by homologous recombination. Cell Res..

[88] d'Adda di Fagagna F. (2014). A direct role for small non-coding RNAs in DNA damage response. Trends Cell Biol..

[89] Michalik K.M., Bottcher R., Forstemann K. (2012). A small RNA response at DNA ends in Drosophila. Nucleic Acids Res..

[90] Francia S., Michelini F., Saxena A., Tang D., de Hoon M., Anelli V., Mione M., Carninci P., d'Adda di Fagagna F. (2012). Site-specific DICER and DROSHA RNA products control the DNA-damage response. Nature.

[91] Castanotto D., Tommasi S., Li M., Li H., Yanow S., Pfeifer G.P., Rossi J.J. (2005). Short hairpin RNA-directed cytosine (CpG) methylation of the RASSF1A gene promoter in HeLa cells. Mol. Ther..

[92] Janowski B.A., Kaihatsu K., Huffman K.E., Schwartz J.C., Ram R., Hardy D., Mendelson C.R., Corey D.R. (2005). Inhibiting transcription of chromosomal DNA with antigene peptide nucleic acids. Nat. Chem. Biol..

[93] Janowski B.A., Huffman K.E., Schwartz J.C., Ram R., Hardy D., Shames D.S., Minna J.D., Corey D.R. (2005). Inhibiting gene expression at transcription start sites in chromosomal DNA with antigene RNAs. Nat. Chem. Biol..

[94] Cho S., Park J.S., Kang Y.K. (2014). AGO2 and SETDB1 cooperate in promoter-targeted transcriptional silencing of the androgen receptor gene. Nucleic Acids Res..

[95] Roberts T.C., Hart J.R., Kaikkonen M.U., Weinberg M.S., Vogt P.K., Morris K.V. (2015). Quantification of nascent transcription by bromouridine immunocapture nuclear run-on RT-qPCR. Nat. Protoc..

[96] Napoli S., Pastori C., Magistri M., Carbone G.M., Catapano C.V. (2009). Promoter-specific transcriptional interference and c-myc gene silencing by siRNAs in human cells. EMBO J..

[97] Zhang M.X., Ou H., Shen Y.H., Wang J., Coselli J., Wang X.L. (2005). Regulation of endothelial nitric oxide synthase by small RNA. Proc. Natl. Acad. Sci. U.S.A..

[98] Hawkins P.G., Santoso S., Adams C., Anest V., Morris K.V. (2009). Promoter targeted small RNAs induce long-term transcriptional gene silencing in human cells. Nucleic Acids Res..

[99] Roberts T.C., Andaloussi S.E., Morris K.V., McClorey G., Wood M.J. (2012). Small RNA-mediated epigenetic myostatin silencing. Mol. Ther. Nucleic Acids.

[100] Weinberg M.S., Villeneuve L.M., Ehsani A., Amarzguioui M., Aagaard L., Chen Z.X., Riggs A.D., Rossi J.J., Morris K.V. (2006). The antisense strand of small interfering RNAs directs histone methylation and transcriptional gene silencing in human cells. RNA.

[101] Suzuki K., Shijuuku T., Fukamachi T., Zaunders J., Guillemin G., Cooper D., Kelleher A. (2005). Prolonged transcriptional silencing and CpG methylation induced by siRNAs targeted to the HIV-1 promoter region. J. RNAi Gene Silencing.

[102] Mendez C., Ahlenstiel C.L., Kelleher A.D. (2015). Post-transcriptional gene silencing, transcriptional gene silencing and human immunodeficiency virus. World J. Virol..

[103] Lim J.W., Snider L., Yao Z., Tawil R., Van Der Maarel S.M., Rigo F., Bennett C.F., Filippova G.N., Tapscott S.J. (2015). DICER/AGO-dependent epigenetic silencing of D4Z4 repeats enhanced by exogenous siRNA suggests mechanisms and therapies for FSHD. Hum. Mol. Genet..

[104] Turunen M.P., Husso T., Musthafa H., Laidinen S., Dragneva G., Laham-Karam N., Honkanen S., Paakinaho A., Laakkonen J.P., Gao E. (2014). Epigenetic upregulation of endogenous VEGF-A reduces myocardial infarct size in mice. PLoS One.

[105] Reebye V., Saetrom P., Mintz P.J., Huang K.W., Swiderski P., Peng L., Liu C., Liu X., Lindkaer-Jensen S., Zacharoulis D. (2014). Novel RNA oligonucleotide improves liver function and inhibits liver carcinogenesis in vivo. Hepatology.

[106] Lee A.Y., Chiang C.M. (2009). Chromatin adaptor Brd4 modulates E2 transcription activity and protein stability. J. Biol. Chem..

[107] Mao C.D., Byers S.W. (2011). Cell-context dependent TCF/LEF expression and function: alternative tales of repression, de-repression and activation potentials. Crit. Rev. Eukaryot. Gene Expr..

[108] Schlereth K., Heyl C., Krampitz A.M., Mernberger M., Finkernagel F., Scharfe M., Jarek M., Leich E., Rosenwald A., Stiewe T. (2013). Characterization of the p53 cistrome–DNA binding cooperativity dissects p53's tumor suppressor functions. PLoS Genet..

[109] Kamachi Y., Kondoh H. (2013). Sox proteins: regulators of cell fate specification and differentiation. Development.

[110] Gow C.H., Guo C., Wang D., Hu Q., Zhang J. (2014). Differential involvement of E2A-corepressor interactions in distinct leukemogenic pathways. Nucleic Acids Res..

[111] Yoshida H., Hirano K., Sato T., Mitsuda N., Nomoto M., Maeo K., Koketsu E., Mitani R., Kawamura M., Ishiguro S. (2014). DELLA protein functions as a transcriptional activator through the DNA binding of the indeterminate domain family proteins. Proc. Natl. Acad. Sci. U.S.A..

[112] Stonestrom A.J., Hsu S.C., Jahn K.S., Huang P., Keller C.A., Giardine B.M., Kadauke S., Campbell A.E., Evans P., Hardison R.C. (2015). Functions of BET proteins in erythroid gene expression. Blood.

[113] Shi Y., Lee J.S., Galvin K.M. (1997). Everything you have ever wanted to know about Yin Yang 1. Biochim. Biophys. Acta.

[114] Weth O., Renkawitz R. (2011). CTCF function is modulated by neighboring DNA binding factors. Biochem. Cell Biol..

[115] Li L.C., Okino S.T., Zhao H., Pookot D., Place R.F., Urakami S., Enokida H., Dahiya R. (2006). Small dsRNAs induce transcriptional activation in human cells. Proc. Natl. Acad. Sci. U.S.A..

[116] Weinberg M.S., Morris K.V. (2013). Long non-coding RNA targeting and transcriptional de-repression. Nucleic Acid Ther..

[117] Huang V., Qin Y., Wang J., Wang X., Place R.F., Lin G., Lue T.F., Li L.C. (2010). RNAa is conserved in mammalian cells. PLoS One.

[118] Wang C., Chen Z., Ge Q., Hu J., Li F., Xu H., Ye Z., Li L.C. (2014). Up-regulation of p21(WAF1/CIP1) by miRNAs and its implications in bladder cancer cells. FEBS Lett..

[119] Wang J., Huang V., Ye L., Barcena A., Lin G., Lue T.F., Li L.C. (2015). Identification of small activating RNAs that enhance endogenous OCT4 expression in human mesenchymal stem cells. Stem Cells Dev..

[120] Chen Z., Place R.F., Jia Z.J., Pookot D., Dahiya R., Li L.C. (2008). Antitumor effect of dsRNA-induced p21(WAF1/CIP1) gene activation in human bladder cancer cells. Mol. Cancer Ther..

[121] Wang J., Place R.F., Huang V., Wang X., Noonan E.J., Magyar C.E., Huang J., Li L.C. (2010). Prognostic value and function of KLF4 in prostate cancer: RNAa and vector-mediated overexpression identify KLF4 as an inhibitor of tumor cell growth and migration. Cancer Res..

[122] Janowski B.A., Younger S.T., Hardy D.B., Ram R., Huffman K.E., Corey D.R. (2007). Activating gene expression in mammalian cells with promoter-targeted duplex RNAs. Nat. Chem. Biol..

[123] Watts J.K., Yu D., Charisse K., Montaillier C., Potier P., Manoharan M., Corey D.R. (2010). Effect of chemical modifications on modulation of gene expression by duplex antigene RNAs that are complementary to non-coding transcripts at gene promoters. Nucleic Acids Res..

[124] Matsui M., Chu Y., Zhang H., Gagnon K.T., Shaikh S., Kuchimanchi S., Manoharan M., Corey D.R., Janowski B.A. (2013). Promoter RNA links transcriptional regulation of inflammatory pathway genes. Nucleic Acids Res..

[125] Matsui M., Sakurai F., Elbashir S., Foster D.J., Manoharan M., Corey D.R. (2010). Activation of LDL receptor expression by small RNAs complementary to a noncoding transcript that overlaps the LDLR promoter. Chem. Biol..

[126] (2003). Whither RNAi?. Nat. Cell Biol..

[127] Weinberg M.S., Barichievy S., Schaffer L., Han J., Morris K.V. (2007). An RNA targeted to the HIV-1 LTR promoter modulates indiscriminate off-target gene activation. Nucleic Acids Res..

[128] Moses J., Goodchild A., Rivory L.P. (2010). Intended transcriptional silencing with siRNA results in gene repression through sequence-specific off-targeting. RNA.

[129] Janowski B.A., Huffman K.E., Schwartz J.C., Ram R., Nordsell R., Shames D.S., Minna J.D., Corey D.R. (2006). Involvement of AGO1 and AGO2 in mammalian transcriptional silencing. Nat. Struct. Mol. Biol..

[130] Chu Y., Yue X., Younger S.T., Janowski B.A., Corey D.R. (2010). Involvement of argonaute proteins in gene silencing and activation by RNAs complementary to a non-coding transcript at the progesterone receptor promoter. Nucleic Acids Res..

[131] Zhang X., Li H., Burnett J.C., Rossi J.J. (2014). The role of antisense long noncoding RNA in small RNA-triggered gene activation. RNA.

[132] Kim D.H., Villeneuve L.M., Morris K.V., Rossi J.J. (2006). Argonaute-1 directs siRNA-mediated transcriptional gene silencing in human cells. Nat. Struct. Mol. Biol..

[133] Morris K.V., Santoso S., Turner A.M., Pastori C., Hawkins P.G. (2008). Bidirectional transcription directs both transcriptional gene activation and suppression in human cells. PLoS Genet..

[134] Huang V., Zheng J., Qi Z., Wang J., Place R.F., Yu J., Li H., Li L.C. (2013). Ago1 Interacts with RNA polymerase II and binds to the promoters of actively transcribed genes in human cancer cells. PLoS Genet..

[135] Toscano-Garibay J.D., Aquino-Jarquin G. (2014). Transcriptional regulation mechanism mediated by miRNA–DNA*DNA triplex structure stabilized by Argonaute. Biochim. Biophys. Acta.

[136] Nakama M., Kawakami K., Kajitani T., Urano T., Murakami Y. (2012). DNA-RNA hybrid formation mediates RNAi-directed heterochromatin formation. Genes Cells.

[137] Zhang Y., Fan M., Zhang X., Huang F., Wu K., Zhang J., Liu J., Huang Z., Luo H., Tao L. (2014). Cellular microRNAs up-regulate transcription via interaction with promoter TATA-box motifs. RNA.

[138] Carninci P., Kasukawa T., Katayama S., Gough J., Frith M.C., Maeda N., Oyama R., Ravasi T., Lenhard B., Wells C. (2005). The transcriptional landscape of the mammalian genome. Science.

[139] Kapranov P., Cheng J., Dike S., Nix D.A., Duttagupta R., Willingham A.T., Stadler P.F., Hertel J., Hackermuller J., Hofacker I.L. (2007). RNA maps reveal new RNA classes and a possible function for pervasive transcription. Science.

[140] Yelin R., Dahary D., Sorek R., Levanon E.Y., Goldstein O., Shoshan A., Diber A., Biton S., Tamir Y., Khosravi R. (2003). Widespread occurrence of antisense transcription in the human genome. Nat. Biotechnol..

[141] Katayama S., Tomaru Y., Kasukawa T., Waki K., Nakanishi M., Nakamura M., Nishida H., Yap C.C., Suzuki M., Kawai J. (2005). Antisense transcription in the mammalian transcriptome. Science.

[142] Morris K.V., Mattick J.S. (2014). The rise of regulatory RNA. Nat. Rev. Genet..

[143] Schwartz J.C., Younger S.T., Nguyen N.B., Hardy D.B., Monia B.P., Corey D.R., Janowski B.A. (2008). Antisense transcripts are targets for activating small RNAs. Nat. Struct. Mol. Biol..

[144] Sanyal A., Lajoie B.R., Jain G., Dekker J. (2012). The long-range interaction landscape of gene promoters. Nature.

[145] Zong X., Huang L., Tripathi V., Peralta R., Freier S.M., Guo S., Prasanth K.V. (2015). Knockdown of nuclear-retained long noncoding RNAs using modified DNA antisense oligonucleotides. Methods Mol. Biol..

[146] Wang Y., Juranek S., Li H., Sheng G., Tuschl T., Patel D.J. (2008). Structure of an argonaute silencing complex with a seed-containing guide DNA and target RNA duplex. Nature.

[147] Bassett A.R., Akhtar A., Barlow D.P., Bird A.P., Brockdorff N., Duboule D., Ephrussi A., Ferguson-Smith A.C., Gingeras T.R., Haerty W. (2014). Considerations when investigating lncRNA function in vivo. Elife.

[148] Dodd D.W., Gagnon K.T., Corey D.R. (2013). Digital quantitation of potential therapeutic target RNAs. Nucleic Acids Ther..

[149] Woolnough J.L., Atwood B.L., Giles K.E. (2015). Argonaute 2 binds directly to tRNA genes and promotes gene repression in cis. Mol. Cell. Biol..

[150] Grewal S.I., Moazed D. (2003). Heterochromatin and epigenetic control of gene expression. Science.

[151] Thomas M.C., Chiang C.M. (2006). The general transcription machinery and general cofactors. Crit. Rev. Biochem. Mol. Biol..

[152] Venters B.J., Pugh B.F. (2009). How eukaryotic genes are transcribed. Crit. Rev. Biochem. Mol. Biol..

[153] Core L.J., Martins A.L., Danko C.G., Waters C.T., Siepel A., Lis J.T. (2014). Analysis of nascent RNA identifies a unified architecture of initiation regions at mammalian promoters and enhancers. Nat. Genet..

[154] Thomas M.C., Chiang C.M. (2005). E6 oncoprotein represses p53-dependent gene activation via inhibition of protein acetylation independently of inducing p53 degradation. Mol. Cell.

[155] Mulligan P., Westbrook T.F., Ottinger M., Pavlova N., Chang B., Macia E., Shi Y.J., Barretina J., Liu J., Howley P.M. (2008). CDYL bridges REST and histone methyltransferases for gene repression and suppression of cellular transformation. Mol. Cell.

[156] Jeon Y., Lee J.T. (2011). YY1 tethers Xist RNA to the inactive X nucleation center. Cell.

[157] Jain A.K., Allton K., Iacovino M., Mahen E., Milczarek R.J., Zwaka T.P., Kyba M., Barton M.C. (2012). p53 regulates cell cycle and microRNAs to promote differentiation of human embryonic stem cells. PLoS Biol..

[158] Wen H., Li Y., Xi Y., Jiang S., Stratton S., Peng D., Tanaka K., Ren Y., Xia Z., Wu J. (2014). ZMYND11 links histone H3.3K36me3 to transcription elongation and tumour suppression. Nature.

[159] Rauwel B., Jang S.M., Cassano M., Kapopoulou A., Barde I., Trono D. (2015). Release of human cytomegalovirus from latency by a KAP1/TRIM28 phosphorylation switch. Elife.

[160] Hou S.Y., Wu S.Y., Zhou T., Thomas M.C., Chiang C.M. (2000). Alleviation of human papillomavirus E2-mediated transcriptional repression via formation of a TATA binding protein (or TFIID)-TFIIB-RNA polymerase II-TFIIF preinitiation complex. Mol. Cell. Biol..

[161] Bourdon J.C., Fernandes K., Murray-Zmijewski F., Liu G., Diot A., Xirodimas D.P., Saville M.K., Lane D.P. (2005). p53 isoforms can regulate p53 transcriptional activity. Genes Dev..

[162] Wang W.M., Wu S.Y., Lee A.Y., Chiang C.M. (2011). Binding site specificity and factor redundancy in activator protein-1-driven human papillomavirus chromatin-dependent transcription. J. Biol. Chem..

[163] Wu S.Y., Chiang C.M. (2007). The double bromodomain-containing chromatin adaptor Brd4 and transcriptional regulation. J. Biol. Chem..

[164] Agalioti T., Lomvardas S., Parekh B., Yie J., Maniatis T., Thanos D. (2000). Ordered recruitment of chromatin modifying and general transcription factors to the IFN-beta promoter. Cell.

[165] Roe J.S., Mercan F., Rivera K., Pappin D.J., Vakoc C.R. (2015). BET Bromodomain Inhibition Suppresses the Function of Hematopoietic Transcription Factors in Acute Myeloid Leukemia. Mol Cell.

[166] Shi J., Wang Y., Zeng L., Wu Y., Deng J., Zhang Q., Lin Y., Li J., Kang T., Tao M. (2014). Disrupting the interaction of BRD4 with diacetylated Twist suppresses tumorigenesis in basal-like breast cancer. Cancer Cell.

[167] Li W., Hu Y., Oh S., Ma Q., Merkurjev D., Song X., Zhou X., Liu Z., Tanasa B., He X. (2015). Condensin I and II complexes license full estrogen receptor alpha-dependent enhancer Activation. Mol. Cell.

[168] Sahu G., Wang D., Chen C.B., Zhurkin V.B., Harrington R.E., Appella E., Hager G.L., Nagaich A.K. (2010). p53 binding to nucleosomal DNA depends on the rotational positioning of DNA response element. J. Biol. Chem..

[169] Kaneko S., Son J., Bonasio R., Shen S.S., Reinberg D. (2014). Nascent RNA interaction keeps PRC2 activity poised and in check. Genes Dev..

[170] Hojfeldt J.W., Van Dyke A.R., Mapp A.K. (2011). Transforming ligands into transcriptional regulators: building blocks for bifunctional molecules. Chem. Soc. Rev..

[171] Jiao A.L., Slack F.J. (2014). RNA-mediated gene activation. Epigenetics.

[172] Liao J.Y., Ma L.M., Guo Y.H., Zhang Y.C., Zhou H., Shao P., Chen Y.Q., Qu L.H. (2010). Deep sequencing of human nuclear and cytoplasmic small RNAs reveals an unexpectedly complex subcellular distribution of miRNAs and tRNA 3′ trailers. PLoS One.

[173] Jeffries C.D., Fried H.M., Perkins D.O. (2011). Nuclear and cytoplasmic localization of neural stem cell microRNAs. RNA.

[174] Roberts T.C. (2014). The MicroRNA biology of the mammalian nucleus. Mol. Ther. Nucleic Acids.

[175] Hwang H.W., Wentzel E.A., Mendell J.T. (2007). A hexanucleotide element directs microRNA nuclear import. Science.

[176] Chu Y., Wang T., Dodd D., Xie Y., Janowski B.A., Corey D.R. (2015). Intramolecular circularization increases efficiency of RNA sequencing and enables CLIP-Seq of nuclear RNA from human cells. Nucleic Acids Res..

[177] Portnoy V., Huang V., Place R.F., Li L.C. (2011). Small RNA and transcriptional upregulation. Wiley Interdiscip. Rev. RNA.

[178] Salmanidis M., Pillman K., Goodall G., Bracken C. (2014). Direct transcriptional regulation by nuclear microRNAs. Int. J. Biochem. Cell Biol..

[179] Place R.F., Li L.C., Pookot D., Noonan E.J., Dahiya R. (2008). MicroRNA-373 induces expression of genes with complementary promoter sequences. Proc. Natl. Acad. Sci. U.S.A..

[180] Kim D.H., Saetrom P., Snove O., Rossi J.J. (2008). MicroRNA-directed transcriptional gene silencing in mammalian cells. Proc. Natl. Acad. Sci. U.S.A..

[181] Huang V., Place R.F., Portnoy V., Wang J., Qi Z., Jia Z., Yu A., Shuman M., Yu J., Li L.C. (2012). Upregulation of Cyclin B1 by miRNA and its implications in cancer. Nucleic Acids Res..

[182] Younger S.T., Corey D.R. (2011). Transcriptional gene silencing in mammalian cells by miRNA mimics that target gene promoters. Nucleic Acids Res..

[183] Naftelberg S., Schor I.E., Ast G., Kornblihtt A.R. (2015). Regulation of alternative splicing through coupling with transcription and chromatin structure. Annu. Rev. Biochem..

[184] Allo M., Buggiano V., Fededa J.P., Petrillo E., Schor I., de la Mata M., Agirre E., Plass M., Eyras E., Elela S.A. (2009). Control of alternative splicing through siRNA-mediated transcriptional gene silencing. Nat. Struct. Mol. Biol..

[185] Allo M., Agirre E., Bessonov S., Bertucci P., Gomez Acuna L., Buggiano V., Bellora N., Singh B., Petrillo E., Blaustein M. (2014). Argonaute-1 binds transcriptional enhancers and controls constitutive and alternative splicing in human cells. Proc. Natl. Acad. Sci. U.S.A..

[186] Ameyar-Zazoua M., Rachez C., Souidi M., Robin P., Fritsch L., Young R., Morozova N., Fenouil R., Descostes N., Andrau J.C. (2012). Argonaute proteins couple chromatin silencing to alternative splicing. Nat. Struct. Mol. Biol..

[187] Batsche E., Ameyar-Zazoua M. (2015). The influence of Argonaute proteins on alternative RNA splicing. Wiley Interdiscip. Rev. RNA.

[188] Agirre E., Bellora N., Allo M., Pages A., Bertucci P., Kornblihtt A.R., Eyras E. (2015). A chromatin code for alternative splicing involving a putative association between CTCF and HP1alpha proteins. BMC Biol..

[189] Arechavala-Gomeza V., Graham I.R., Popplewell L.J., Adams A.M., Aartsma-Rus A., Kinali M., Morgan J.E., van Deutekom J.C., Wilton S.D., Dickson G. (2007). Comparative analysis of antisense oligonucleotide sequences for targeted skipping of exon 51 during dystrophin pre-mRNA splicing in human muscle. Hum. Gene Ther..

[190] Aartsma-Rus A., Janson A.A., Kaman W.E., Bremmer-Bout M., den Dunnen J.T., Baas F., van Ommen G.J., van Deutekom J.C. (2003). Therapeutic antisense-induced exon skipping in cultured muscle cells from six different DMD patients. Hum. Mol. Genet..

[191] van Deutekom J.C., Janson A.A., Ginjaar I.B., Frankhuizen W.S., Aartsma-Rus A., Bremmer-Bout M., den Dunnen J.T., Koop K., van der Kooi A.J., Goemans N.M. (2007). Local dystrophin restoration with antisense oligonucleotide PRO051. N. Engl. J. Med..

[192] Voit T., Topaloglu H., Straub V., Muntoni F., Deconinck N., Campion G., De Kimpe S.J., Eagle M., Guglieri M., Hood S. (2014). Safety and efficacy of drisapersen for the treatment of Duchenne muscular dystrophy (DEMAND II): an exploratory, randomised, placebo-controlled phase 2 study. Lancet Neuro.l.

[193] Jarver P., O'Donovan L., Gait M.J. (2014). A chemical view of oligonucleotides for exon skipping and related drug applications. Nucleic Acids Ther..

[194] Kang S.H., Cho M.J., Kole R. (1998). Up-regulation of luciferase gene expression with antisense oligonucleotides: implications and applications in functional assay development. Biochemistry.

[195] Liu J., Hu J., Corey D.R. (2012). Expanding the action of duplex RNAs into the nucleus: redirecting alternative splicing. Nucleic Acids Res..

[196] Yu D., Sakurai F., Corey D.R. (2011). Clonal Rett Syndrome cell lines to test compounds for activation of wild-type MeCP2 expression. Bioorg. Med. Chem. Lett..

[197] Liu J., Hu J., Hicks J.A., Prakash T.P., Corey D.R. (2015). Modulation of splicing by single-stranded silencing RNAs. Nucleic Acids Ther..

[198] Taliaferro J.M., Aspden J.L., Bradley T., Marwha D., Blanchette M., Rio D.C. (2013). Two new and distinct roles for Drosophila Argonaute-2 in the nucleus: alternative pre-mRNA splicing and transcriptional repression. Genes Dev..

